# Efficacy and long-term outcomes of congenital penile curvature treatment in children using tunica albuginea plication and Heinecke–Mikulicz corporoplasty

**DOI:** 10.1007/s00383-026-06302-z

**Published:** 2026-02-03

**Authors:** Aziz Serhat Baykara, Cigdem Arslan Alici

**Affiliations:** 1 Department of Pediatric Surgery, Eskisehir City Hospital, University of Health Sciences, 71 Evler Neighborhood, Cavdarlar Street, Odunpazari, Eskisehir, 26080 Turkey; 2 Department of Pediatric Urology, Eskisehir City Hospital, University of Health Sciences, Eskisehir, Turkey

**Keywords:** Congenital penile curvature, Penile degloving, Tunica albuginea plication, Heinecke-Mikulicz corporoplasty

## Abstract

**Purpose:**

This study aimed to compare the efficacy and long-term outcomes of tunica albuginea plication and Heinecke–Mikulicz corporoplasty techniques in children.

**Methods:**

A retrospective review was performed on 176 children who underwent corrective surgery for CPC with a curvature angle between 30° and 60° between 2014 and 2022. Patients were classified into three groups: degloving alone (Group 1, *n* = 85), tunica albuginea plication (Group 2, *n* = 72), and Heinecke–Mikulicz corporoplasty (Group 3, *n* = 19).

**Results:**

The mean age at surgery was 7.6 ± 3.2 years (range: 4–11). Curvature was mainly ventral or ventrolateral (84.8%). Mean preoperative curvature angles were 36.2°, 37.4°, and 41.4° in Groups 1, 2, and 3, respectively. Adequate correction was achieved with degloving alone in 48.2% of patients. In those requiring additional correction, complete straightening was obtained in all cases (*p* < 0.001). During a mean follow-up of 26.7 ± 17.7 months, curvature recurrence occurred in 18.8%, 19.4%, and 21.1% of patients in Groups 1, 2, and 3, respectively. Late complications included palpable suture knots in two patients from Group 2 and four from Group 3.

**Conclusions:**

Tunica albuginea plication is a safe, effective, and practical option for correcting penile curvature in children when degloving alone is insufficient.

## Introduction

Congenital penile curvature (CPC) refers to penile deformities present from birth that, if left untreated, can lead to significant sexual and psychological problems later in life. Its epidemiology remains unclear due to the lack of large-scale studies and the exclusion of mild penile curvatures (less than 20°) from statistics. According to the Birth Defects Monitoring Program (BDMP, USA) and other studies, the incidence may range between 4% and 10% [1]. The etiology of CPC is thought to involve cutaneous or subcutaneous chords, disproportion of the corpus cavernosum, and, rarely, a short urethra [2]. It has also been suggested that this congenital penile anomaly may result from androgen deficiency or reduced androgen sensitivity during fetal development [3].

Diagnosis of penile curvature is based on patient history and assessment of the penis in the erect state, either through photographs taken in at least three projections or via pharmacologically induced erection in the hospital [4]. Penile curvature is classified according to its severity as insignificant (< 20°), mild (20–30°), moderate (30–60°), or severe (> 60°) [5]. Currently, early surgical correction of penile curvature is recommended to ensure healthy psychosocial development in children [6–8]. Several surgical techniques have been described for the correction of penile curvature. The fundamental initial approach involves deepening an incision through the superficial Buck fascia and degloving the penis to achieve orthoplasty [1]. For persistent curvature after penile degloving, various procedures have been proposed, including excisional corporoplasty (e.g., Nesbit procedure and Kelami modification), incisional Heinecke-Mikulicz (H-M) corporoplasty, incisionless tunica albuginea plication (TAP), penile disassembly techniques, and penile grafting procedures [9]. However, the optimal surgical technique and timing remain a subject of debate, and long-term follow-up data are limited.

The aim of this study is to evaluate and compare the effectiveness of TAP and H-M corporoplasty techniques in children with moderate penile curvature (30–60°), including those who demonstrated improvement after degloving.

## Materials and methods

### Study design and ethical considerations

We retrospectively included patients who underwent surgery for isolated congenital penile curvature (CPC) between 30° and 60° from January 2014 to September 2022. Ethical approval was obtained from the University of Health Sciences, Eskisehir City Hospital Scientific Research Ethics Committee (ESH/GOEK 2023/60, 22/11/2023). Written informed consent was obtained from all participants prior to enrollment. All patients were thoroughly informed about the planned surgical procedure, including potential risks and complications.

Patients with a curvature degree below 30° or above 60°, those with additional penile anomalies (such as hypospadias, epispadias, or bladder exstrophy), and patients with a history of prior penile curvature surgery were excluded from the study. Patients who underwent surgery for isolated CPC were divided into three groups: those whose curvature was corrected after penile degloving (Group 1, *n* = 85), those who underwent tunica albuginea plication (TAP) procedure (Group 2, *n* = 72), and those who underwent Heinecke–Mikulicz corporoplasty (Group 3, *n* = 19). Successful correction was defined as a postoperative penile curvature of less than 10° in all groups.

All patients were discharged on the second postoperative day following removal of the penile dressing and urethral catheter. The first follow-up visit was scheduled at three weeks postoperatively, and subsequent follow-ups were conducted at three-month intervals. Patient records and postoperative follow-up notes were thoroughly reviewed, and relevant data were collected. Since our patients had not yet reached sexual activity, no subjective scales were used to assess patient satisfaction.

### Surgical techniques

Following anesthesia induction, all patients received intravenous antibiotic prophylaxis and a urethral catheter was inserted. A rubber Penrose drain was tied at the base of the penis to apply a penile tourniquet. Subsequently, a saline solution was injected into the proximal one-third of the corpora cavernosa to induce an artificial erection. During artificial erection, penile length was measured using a rigid ruler, and the degree and direction of penile curvature were determined using a goniometer and recorded. Patients with a curvature of less than 30° or greater than 60° were excluded from the study. In patients with a curvature degree between 30 and 60 degrees, the penis was circumferentially degloved below the coronal level, and the neurovascular bundle was dissected. During degloving, superficial fibrotic bands and adhesions were released, and the penis was completely degloved. If the curvature was corrected after degloving, circumcision was performed, and the operation was concluded.

**Fig. 1:**
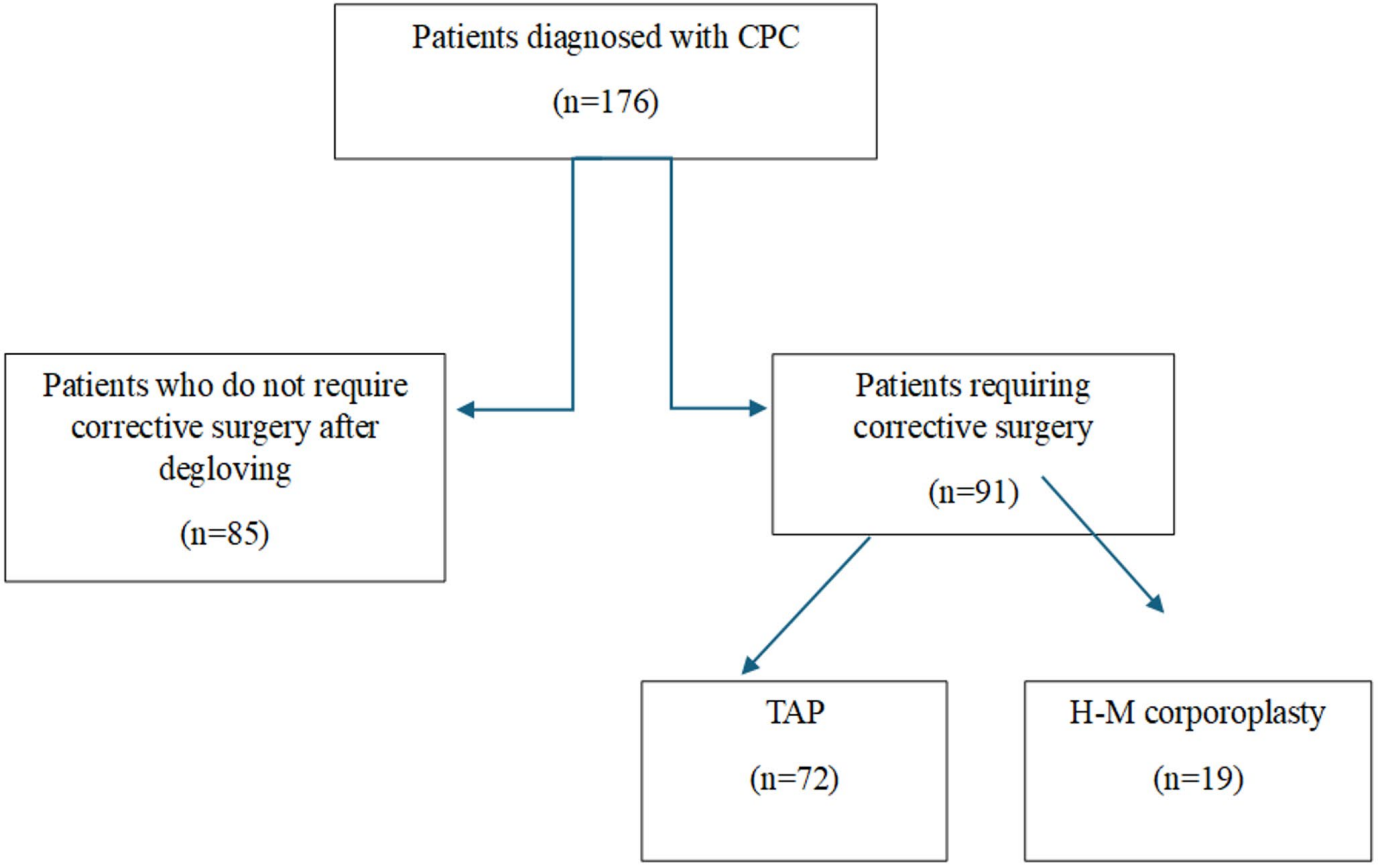
Diagram of the surgical approach for patients with isolated CPC

In cases where degloving alone did not achieve adequate penile straightening, correction was performed using either tunica albuginea plication (TAP) or Heinecke–Mikulicz (H-M) corporoplasty (Fig. 1). No patient underwent both techniques simultaneously. The incisions for both TAP and H-M corporoplasty were placed as described by Baskin, i.e., in the midline over the convex side of the curvature. In all patients, plication or corporoplasty was performed only on the convex side, and no bilateral corporotomies were made.

**Fig. 2:**
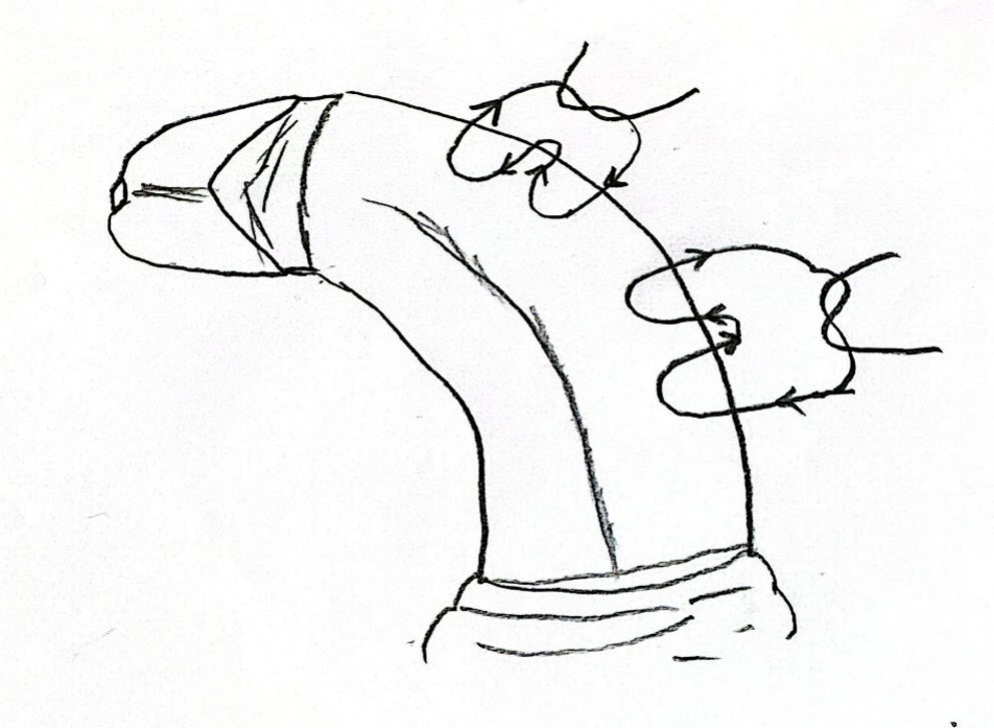
Schematic technical illustration of the tunica albuginea plication (TAP) technique showing the placement of plication sutures

For TAP, the dorsal neurovascular bundle was mobilized only to the extent necessary to permit safe placement of dorsal plication sutures and to minimize the risk of neurovascular injury; no deep dorsal incision was performed. After induction of an artificial erection, residual penile curvature was assessed intraoperatively, and the number of plication sutures was determined accordingly. In all patients, plication was performed on the convex side of the curvature. In cases in which dorsal tunica albuginea plication was performed, a total of one to three absorbable dorsal plication sutures 5 − 0 polydioxanone (PDS) were placed, each measuring approximately 8–12 mm in length, depending on the severity of the curvature (Fig. 2). The sutures were placed symmetrically along the dorsal midline of the tunica albuginea at the point of maximal curvature. Adequacy of curvature correction was confirmed by repeating the artificial erection.

**Fig. 3:**
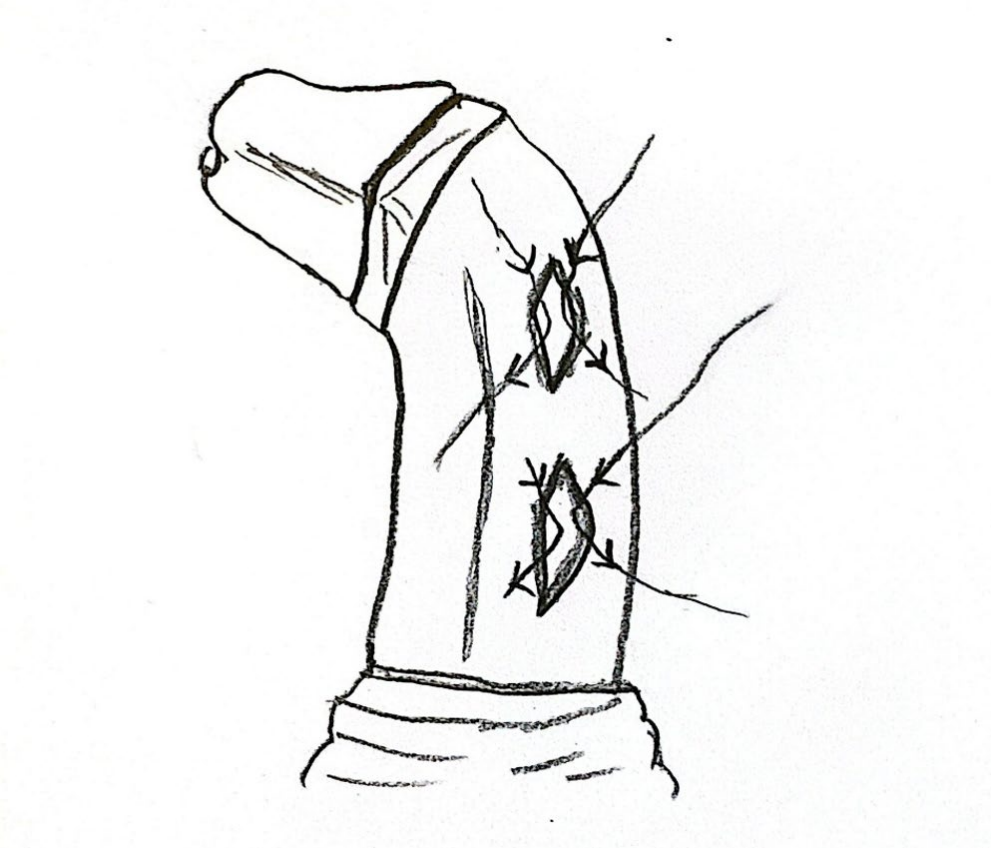
Technical drawing of the Heinecke–Mikulicz corporaplasty showing longitudinal incision of the tunica albuginea followed by transverse closure for penile straightening

H–M corporoplasty was performed in patients with moderate penile curvature ranging between 30° and 60°. The dorsal neurovascular bundle was gently mobilized only to the extent necessary to ensure safe suture placement and to prevent inadvertent injury during manipulation of the tunica albuginea. Longitudinal ventral tunical incisions were made at the point of maximal curvature on the concave side of the penis. Depending on the severity of the curvature, one to three incisions were performed. Each incision measured approximately 8–12 mm in length and was subsequently closed transversely using the H–M technique with 5.0 absorbable PDS sutures to achieve curvature correction (Fig. 3). The adequacy of correction was confirmed by inducing a repeat artificial erection.

Following the corrective surgical procedures described above, the neurovascular bundle was repositioned, and the Buck fascia was closed. Circumferential penile skin was sutured, and the procedure was completed. A moderate, distal-to-proximal, rolled gauze bandage was applied to the penis. All patients were discharged after removal of the penile bandage on postoperative day 2. To prevent reflex erections postoperatively, oxybutynin hydrochloride syrup was administered for one month [10]. The surgical approach for patients with isolated CPC is summarized in Fig. 3.

### Statistical analysis

Statistical analyses were performed using IBM SPSS Statistics for Windows, Version 26.0 (IBM Corp., Armonk, NY, USA). The normality of data distribution was assessed using the Shapiro–Wilk test. Pearson’s correlation coefficient was used for normally distributed variables, and Spearman’s rank correlation coefficient was applied for non-normally distributed variables. Associations between categorical variables were evaluated using the Chi-square test or Fisher’s exact test when appropriate. Quantitative data were presented as mean ± standard deviation (SD) or median (Q1–Q3), and qualitative data as frequency (n) and percentage (%). A p-value < 0.05 was considered statistically significant.

## Results

A total of 176 patients with isolated congenital penile curvature (CPC) underwent curvature correction surgery. The mean age of the patients was 7.6 ± 3.21 years (range: 4–11 years). The curvature direction was ventral or ventrolateral in the majority of patients (84.8%). The mean operative time was 32 ± 3.12 min in the degloving group, 44 ± 4.12 min in the TAP group, and 42 ± 3.16 min in the H-M corporoplasty group. Following degloving, penile curvature was corrected in 48.2% of patients. Patients with persistent curvature after degloving underwent corrective surgery using either the TAP or H-M corporoplasty techniques. Adequate penile straightening was achieved in both groups undergoing corrective procedures (*p* < 0.001). No statistically significant differences were observed in penile length measurements before and after surgery (*p* < 0.001). None of the patients experienced urethral injury. Preoperative and postoperative penile length and curvature angles are summarized in Table 1.

In the early postoperative period, hematoma was observed in 7 patients and wound infection in 3 patients in Group 1. In Group 2, hematoma occurred in 5 patients and wound infection in 4 patients. In Group 3, hematoma and wound infection were observed in 4 and 1 patient, respectively. The clinical characteristics of the patients are presented in Table 2.

The mean follow-up duration was 26.68 ± 17.70 months (range: 9–57 months). In the late postoperative period, palpable suture knots were detected in 2 patients in Group 2 and 4 patients in Group 3, and these patients were followed up. The first recurrence was observed at 3 months postoperatively. During follow-up, recurrence of curvature greater than 30° was observed in 16 patients (18.8%) in the degloving-only group, 14 patients (19.4%) in the TAP group, and 4 patients (21.1%) in the H-M corporoplasty group. All patients with recurrence exceeding 30° were planned for corrective surgery in the postpubertal period. None of the patients reported erectile dysfunction; however, all patients with recurrent curvature expressed dissatisfaction with the appearance of their penis.

**Table 1:** Comparison of penile curvature angles and erect penile lengths in pre- and postoperative groups

Groups	At baseline	Postoperative	*P* value
Group 1: Degloving (*n* = 85)			
Length of erect penis (cm)	8.4 (± 1.1)	8.8 (± 1.4)	0.119
The angle of the penile curvature (degrees)	36.2 (± 3.22)	6.1 (± 4.2)	< 0.001
Group 2: TAP (*n* = 72)			
Length of erect penis (cm)	8.2 (± 1.2)	7.4 (± 1.2)	0.427
The angle of the penile curvature (degrees)	37.4 (± 4.16)	5.2 (± 4.6)	< 0.001
Group 3: H-M corporoplasty (*n* = 19)			
Length of erect penis (cm)	8.5 (± 1.4)	7.2 (± 1.6)	0.585
The angle of the penile curvature (degrees)	41.4 (± 3.76)	6.8 (± 1.8)	< 0.001

*TAP*  Tunica albuginea plication;* H-M* Heinecke-Mikulicz corporoplasty

**Table 2:** Clinical characteristics of patients with isolated CPC undergoing surgical correction

Characteristics	Group 1: Degloving (*n* = 85)	Group 2: TAP (*n* = 72)	Group 3: H-M (*n* = 19)
Age at Surgery (y)	7.4 ± 3.6	7.6 ± 3.2	7.8 ± 2.4
Operative time (min)	31 ± 2.88	44 ± 4.12	42 ± 3.16
Early and long-term complications			
Hematoma	7 (%8.2)	5 (%6.9)	4 (%21)
Wound infection/abscess	3 (%3.5)	4 (%5.5)	1 (%5.2)
Palpable suture knot	0	2 (%2.7)	4 (%21)
Recurrence of curvature	16 (%18.8)	14 (%19.4)	4 (%21)
Permanent loss of glans sensation	0	0	0
Urethral injury	0	0	0

Values are presented as mean ± standard deviation or number (%). Percentages may not sum to 100% due to rounding

*TAP* tunica albuginea plication;* H-M* Heinecke-Miculicz;* y* year;* min* minute

## Discussion

Normal external genitalia are essential for healthy psychosexual development, and delays in the diagnosis or treatment of CPC can negatively affect psychosocial outcomes and quality of life [11]. The European Association of Urology (EAU) Pediatric Urology Guidelines recommend surgical correction for curvatures exceeding 30°, regardless of age [6, 7]. Similarly, surveys among pediatric urologists indicate that surgical correction should not be delayed in children with 20–30° curvature whose penile appearance during erection is considered abnormal [8]. In children, urinary deviation or penile deviation during morning erections are common reasons for presentation. In societies with routine circumcision, penile abnormalities are often detected by physicians during the procedure. Most cases in our series were identified either by parents or during routine circumcision.

Histopathological studies suggest that isolated CPC results from abnormal tunica albuginea development and increased elasticity on the longer side, rather than urethral anomalies [12]. Consistent with the literature, 84.8% of our patients had ventral or ventrolateral curvature [13].

There is no consensus or established algorithm regarding the surgical technique or timing for patients diagnosed with CPC. However, based on the assumption that delayed diagnosis may lead to adverse outcomes, correction of penile abnormalities before the onset of sexual awareness is recommended [14]. Various methods exist for correcting isolated CPC, yet none are considered the gold standard. In developed countries, penile curvatures are generally diagnosed and treated after puberty; however, in societies where circumcision is common, these abnormalities are often detected and treated earlier. Curvatures identified through family history or pre-circumcision genital examination can be corrected during the same session as the circumcision. Most previous studies have involved small retrospective cohorts treated after puberty. In contrast, our study focused on standardizing the surgical approach for penile curvatures identified before puberty and included long-term postoperative follow-up outcomes.

In the literature, 36–39% of patients with isolated congenital penile curvature (CPC) are reported to have a skin cord [15]. In such cases, curvature can often be corrected by releasing the penile skin through a degloving procedure. Previous studies have demonstrated that penile curvature was corrected in 43–53% of isolated CPC cases following degloving [16]. In our series, degloving alone resulted in correction of curvature in 48.2% of patients, which is consistent with these reported outcomes. However, during follow-up, recurrence of curvature was observed in 18.8% of patients whose curvature initially improved, highlighting that while degloving can be effective, it may not provide a definitive solution in all cases.

In patients whose curvature did not improve following degloving, various corrective surgical techniques based on either incision or excision of the tunica albuginea have been described. The first plication technique for curvature correction was introduced by Nesbit [17]. The Nesbit corporoplasty involves excising ellipsoid areas on the convex surface of the penis and closing these defects with delayed-absorbable monofilament sutures. Despite its high success rates, the Nesbit procedure has been associated with complications such as hematoma, penile numbness, erectile dysfunction, residual curvature, and penile shortening, prompting the development of alternative techniques. To reduce the risk of neurovascular injury, the Heinecke-Mikulicz (H-M) corporoplasty was introduced, which is based on a longitudinal incision and transverse closure of the healthy convex tunica [18]. This approach renders the neurovascular structures less vulnerable during surgery and is associated with a lower complication risk. In our series, we employed the H-M corporoplasty, which we considered less invasive and associated with a lower risk of neurovascular injury. Previous study applying this technique to five patients with curvatures ranging from 30° to 80° reported complete correction of the curvature without complications [16]; however, follow-up duration and recurrence rates were not detailed. In our pediatric cohort, intraoperative penile straightening was achieved in all patients undergoing H-M corporoplasty in a single session, and long-term follow-up revealed sustained adequate penile straightening in 78.9% of patients.

In 1985, Essed and Schroder described the TAP technique without tissue excision or incision [19]. This plication corporoplasty technique is based on suturing the convex side of the tunica without making any incisions or excisions. Subsequently, it was demonstrated that identifying the penile neurovascular anatomy and performing corporoplasty techniques accordingly can prevent neurovascular injury [20]. Consequently, penile degloving combined with TAP gradually gained popularity among pediatric urologists for the correction of isolated CPC. However, a disadvantage of this method is that the sutures may tear the tunica during erections before complete healing, and knots may remain palpable under the skin. In a study assessing the long-term efficacy of dorsal plication in 83 patients, recurrence of curvature was observed in only 13 cases (16%) [21]. In another study including 43 pediatric patients who underwent TAP, recurrence of curvature was reported in 13.9% of patients over a 16-month follow-up [22]. Previous studies have reported favorable outcomes of dorsal plication in the pediatric age group; however, follow-up data beyond puberty are limited. In another study involving 13 pediatric patients who underwent TAP during the prepubertal period, recurrent curvature was detected in seven patients after puberty [2]. According to the literature, the recurrence rate after CPC surgery differs significantly between children and adults. pre‑pubertal patients exhibit a higher risk of recurrence, ranging from 5% to 30% in most series, and up to 54% in some reports [2]. In our study, recurrent curvature requiring surgery was observed in 19.4% of patients who underwent TAP. It remains unclear whether this outcome is related to the technique itself or to the ongoing nature of penile development. Although patients requiring more sutures to achieve a straight penis intraoperatively appeared more prone to recurrence, this observation is insufficient to make a definitive conclusion. In our study, patients who underwent TAP developed less recurrence of curvature. The approach to residual penile curvature is based on the resection of scar tissue followed by plication or dermal grafting [19]. Patients with residual curvature in our study were placed under follow-up, and secondary surgical intervention was planned for the postpubertal period.

Early complications of curvature correction most commonly include hematoma (6.5%) and wound infection (1.4%) [9]. In our study, early postoperative complications, such as hematoma, were consistent with the literature and were managed conservatively without the need for surgical intervention. The infections observed in our series were minor and superficial, likely related to transient local hematoma formation or skin manipulation during the degloving process rather than to deep surgical site contamination. All infections were successfully treated with oral antibiotics without any long-term sequelae. Strict perioperative sterile precautions and meticulous postoperative care were applied to minimize the risk of infection.

Regardless of the technique used for the surgical correction of isolated CPC, three additional postoperative concerns have been described. These include postoperative penile shortening, reduced glans penile sensation (usually transient), and palpable suture knots or granulomas [23]. Penile shortening is considered more a consequence of any plication technique rather than a true complication. The degree of penile shortening is influenced by the direction (more frequently ventral curvatures) and severity of the curvature [24]. In our cohort, no statistically significant penile shortening was observed based on pre- and postoperative measurements.

Mobilization of the neurovascular bundle is considered responsible for transient postoperative sensory reduction in the glans penis. Glans hypoesthesia may reduce sensation during sexual activity; however, even when present, it generally does not impair sexual function [25]. To avoid mobilization of the neurovascular bundle, many surgeons have modified the Nesbit technique. Leonardo et al. [26] evaluated 31 patients with CPC over a mean follow-up of 39 months, comparing outcomes of the Nesbit procedure with incisionless plication. According to their study, glans paresthesia was reported in 75% of patients who underwent the Nesbit procedure, whereas this rate was 37% in the plication group. In our series, postoperative penile paresthesia was observed in all affected patients but was transient, lasting from a few days up to three months.

A common, albeit relatively bothersome, consequence of all plication techniques is the presence of a palpable suture knot at the plication site postoperatively. Although nearly all patients can feel the suture in the area, it is generally not distressing. Studies comparing Nesbit to incisionless plication have reported that patients in the TAP group experienced a higher rate of suture palpation and discomfort during erection, indicating that they were more likely to complain about palpable sutures (approximately 21%) [27]. Contrary to this presentation, in our series, postoperative follow-up revealed more palpable suture knots in patients who underwent H-M corporoplasty. The tunical tissue exhibits a pronounced inflammatory response to foreign materials, which can lead to granuloma formation at the suture site [28]. Therefore, even when absorbable sutures are used, palpable knots may still occur postoperatively. In adults, one study demonstrated that the use of absorbable suture material did not affect the recurrence of curvature in patients undergoing incisional corporoplasty, whereas in those undergoing incisionless corporoplasty, the risk of recurrence increased [29]. In our study, considering that both corporoplasty techniques were applied in a pediatric population and recognizing the potential development of granulomas, palpable sutures, and pain during erection if non-absorbable sutures were used, we opted for absorbable sutures. Another important aspect is postoperative erectile function. Questionnaires such as the International Index of Erectile Function or the Self-Esteem and Relationship Questionnaire were not applicable in our cohort, as the mean age of first sexual intercourse in boys in our country is 17 years [30].

This study has several limitations, including its retrospective design, single-center setting, and a mean follow-up period of less than five years. Future prospectively designed, comparative, and longitudinal studies are needed to confirm these findings and establish more robust conclusions.

Based on the evaluation of both early and late postoperative complications, the TAP technique demonstrates superior feasibility, safety, and efficacy in pediatric patients. Given the critical role of normal-appearing external genitalia in healthy psychosexual development, timely surgical correction is essential. Accordingly, we advocate that corrective surgery for children with isolated CPC should be performed without unnecessary delay to optimize both functional and psychosocial outcomes.

## Data Availability

No datasets were generated or analysed during the current study.
